# Surveillance of clinical research integrity in medically assisted reproduction: a systematic review of retracted publications

**DOI:** 10.3389/fpubh.2023.1210951

**Published:** 2023-08-01

**Authors:** Sabrina Minetto, Mara Zanirato, Sofia Makieva, Daria Marzanati, Stefania Esposito, Valerio Pisaturo, Mauro Costa, Massimo Candiani, Enrico Papaleo, Alessandra Alteri

**Affiliations:** ^1^Obstetrics and Gynaecology Unit, IRCCS San Raffaele Scientific Institute, Milan, Italy; ^2^Kinderwunschzentrum, Klinik für Reproduktions-Endokrinologie, Universitätsspital Zürich, Zürich, Switzerland; ^3^Reproductive Sciences Laboratory, IRCCS San Raffaele Scientific Institute, Milan, Italy; ^4^Reproductive Medicine Department, International Evangelical Hospital, Genoa, Italy; ^5^Faculty of Medicine and Surgery, Vita-Salute San Raffaele University, Milan, Italy

**Keywords:** article retraction, research misconduct, research integrity, medically assisted reproduction, MAR

## Abstract

**Background and purpose:**

Retraction is a significant consequence of scientific research, resulting from various factors ranging from unintentional errors to intentional misconduct. Previous reviews on retracted publications in obstetrics and gynecology have identified “article duplication,” “plagiarism,” and “fabricated results” as the main reasons for retraction. However, the extent of retracted articles in the literature on medically assisted reproduction (MAR) remains unclear. This systematic review aimed to assess the number and characteristics of retracted articles in the field of MAR.

**Methods:**

The Preferred Reporting Items for Systematic Reviews and Meta-Analyses (PRISMA) guidelines were followed for this study. A comprehensive literature search was conducted on the PubMed database from 1993 to February 2023, limited to English articles and including all 283 terms from the International Glossary on Infertility and Fertility Care. To identify retracted studies, a specific query combining the 283 terms from the glossary with a retraction-related keyword was used. Only studies focused on MAR and involving human subjects were included.

**Results:**

The electronic search yielded a total of 523,067 records in the field of infertility and fertility care. Among these, a total of 2,458 records were identified as retracted. The citation retraction rate was found to be 0.47% (2,458/523,067; 95%CI 0.45–0.49), and the citation retraction rate for randomized controlled trials (RCTs) was 0.20% (93/45,616; 95%CI 0.16–0.25). A total of 39 retracted articles specifically related to MAR were identified. Among these, 41.0% were RCTs (*n* = 16), 15.4% were reviews (*n* = 6), and 10.3% were retrospective studies (*n* = 4) or prospective studies (*n* = 4). Most of the retractions occurred shortly after publication, with “plagiarism” being the most common reason for retraction, followed by “duplicate publication.”

**Discussion:**

The issue of retraction exists within the field of infertility and fertility care, including MAR. Our findings indicate that scientific misconduct, particularly plagiarism and duplicate publication, are the primary causes of retraction in MAR. Despite finding that the proportion of retracted citations is low, promoting scientific integrity should be a priority. The consequences of article retractions have significant implications for patient care and the scientific community. Hence, it is crucial to prioritize thorough screening of manuscripts before publication to maintain research integrity.

**Systematic review registration:**

https://www.crd.york.ac.uk/prospero/display_record.php?RecordID=185769, PROSPERO, identifier: CRD42020185769.

## Introduction

Retraction of flawed publications is a measure that safeguards scientific literature, assuring readers the accuracy of published data and the validity of the conclusions. Journal editors, peer reviewers, and the authors themselves are called to responsibly evaluate the studies prior to publication.

The retraction represents one of the most serious penalties in scientific research, used to punish serious violations, such as plagiarism, data falsification or fabrication (image manipulation), undisclosed conflict of interest, lack of ethical approval, fraud or suspected fraud, errors (miscalculation or experimental errors) and redundant publications. In 2019, the Committee for Publication Ethics (COPE) updated their guidelines, redefining standardized criteria for manuscript retraction, aiding editors and all those involved in these processes (1). According to COPE, the publication retraction should be carried out and followed by an editors' retraction notice, which should contain the title, the authors' names and the retraction reason. The retraction notice should be promptly published and linked to the original retracted article, minimizing harmful side effects. Notably, the retraction process has to be handled carefully; as mentioned in COPE, if a small part of an article contains inaccurate data or contents, a correction can be used to rectify the publication.

Several studies in this area, have suggested errors (2–7) as primary causes for manuscript retraction. Furthermore, the number of scientific retractions has increased in recent years and the retraction rate shows a strong correlation with the impact factor (IF) of the journal. Indeed, an article published in a higher-impact journal seems more likely to be retracted than an article published in a lower impact journal (8). Moreover, it has been observed that IF is higher among papers retracted for fraud than among those retracted for error, indicating that authors of fraudulent retracted studies publish in journals of high IF (9).

Accordingly, in the field of gynecology and obstetrics, this topic is becoming increasingly relevant. Chambers and coworkers identified 176 retracted articles in obstetrics and gynecology literature highlighting plagiarism (22.7%) and data falsification (21.0%) as the main reasons for article retraction (10). However, the reasons for retraction were not disclosed in the retraction notices of the included studies and, hence, these percentages should be taken with a pinch of salt (11). A later thorough retrospective review demonstrated that the most common reasons for article retractions in obstetric and maternal-fetal medicine were article duplication (21.3%) and plagiarism (18.9%) (12).

Although Chambers and co-workers identified 16 of the 176 retracted studies belonging to the field of reproductive endocrinology and infertility (10), the number of retracted articles in the field of medically assisted reproduction (MAR) remains unclear, suggesting that, at present, and this thorny topic deserves further attention.

Therefore, the aim of this systematic review was to investigate the number of research retractions in the field of MAR, specifically examining their characteristics with particular emphasis on trends and reasons for retraction.

## Methods

### Systematic review protocol and registration

The study was conducted based on Preferred Reporting Items for Systematic Reviews and Meta-Analyses (PRISMA) guidelines (13). The protocol was registered on the International Prospective Register of Systematic Review (PROSPERO; Registration no. CRD42020185769) (14).

### Search strategy, eligibility criteria, and study selection

Initially, a literature search was conducted from 1993 to February 2023, and only English records were identified using all 283 terms listed in the International Glossary on Infertility and Fertility Care (15), using R Statistical software (R version 4.2.2) with RISmed R package (16, 17). This approach was undertaken to ascertain the estimate of the citation retraction rate of published studies on infertility and fertility care available in the electronic bibliographic database PubMed (Supplementary Table I). The term 'citation retraction rate' was used to refer to the rate of retracted citations among the records identified through the literature search. In this context, we use the term citations to define records, extracted using specific queries (Supplementary Table I), that have not been screened individually due to high volume of literature search results.

Subsequently, in order to perform our systematic review and identify retracted studies, a bibliographical search was conducted using this query “Term[Title/Abstract] AND ((retracted publication[Publication Type]) OR (retraction of publication[Publication Type])) AND English[lang]“ in which the keywords were all 283 terms listed in the International Glossary on Infertility and Fertility Care (15) (Supplementary Table I). Following the search, duplicates were removed. Two criteria for inclusion were determined, which all had to be fulfilled: (i) studies focused on clinical and laboratory practices of MAR; (ii) all types of studies involving humans and related to MAR were included. Unrelated articles, as well as retracted articles related to other different contexts, were excluded. Additionally, basic research studies involving human samples and animal models were excluded.

Two investigators (MZ and VP) independently reviewed titles, abstracts, and full text articles, and selected the studies. Any disagreements were resolved by discussion with a third author (SMi) until a consensus was reached.

We used descriptive statistics to characterize retracted MAR publications. No risk of bias was undertaken as the scope of this review was to report the number and characteristics of retracted MAR articles and not to assess the quality of the studies.

### Data extraction

Each retracted paper was thoroughly investigated and the following data were extracted from each study: (i) Title (PMID and DOI); (ii) author characteristics (number of authors per paper, affiliation, and geographical origin); (iii) journal and journal IF; (iv) type of study; (v) publication date; (vi) retraction date; (vii) number of pre-retraction and post-retraction citation; (viii) retraction reason, and (ix) the authors of the retraction notice.

## Results

A total of 523,067 unique records in the field of infertility and fertility care were obtained from the electronic search, excluding any duplicate entries. Among these, 3,169 records were identified as retracted. After removing 711 duplicates, we identified 2,458 retracted studies. The citation retraction rate of included records was 0.47% (2,458/523,067; 95% CI 0.45–0.49). To determine the citation retraction rate of included records specifically for randomized controlled trials (RCTs), we identified 93 retracted RCTs out of a total of 45,616 RCTs in the field of infertility and fertility care, resulting in an RCT retraction rate of 0.20% (93/45,616; 95% CI 0.16–0.25).

**Figure 1:**
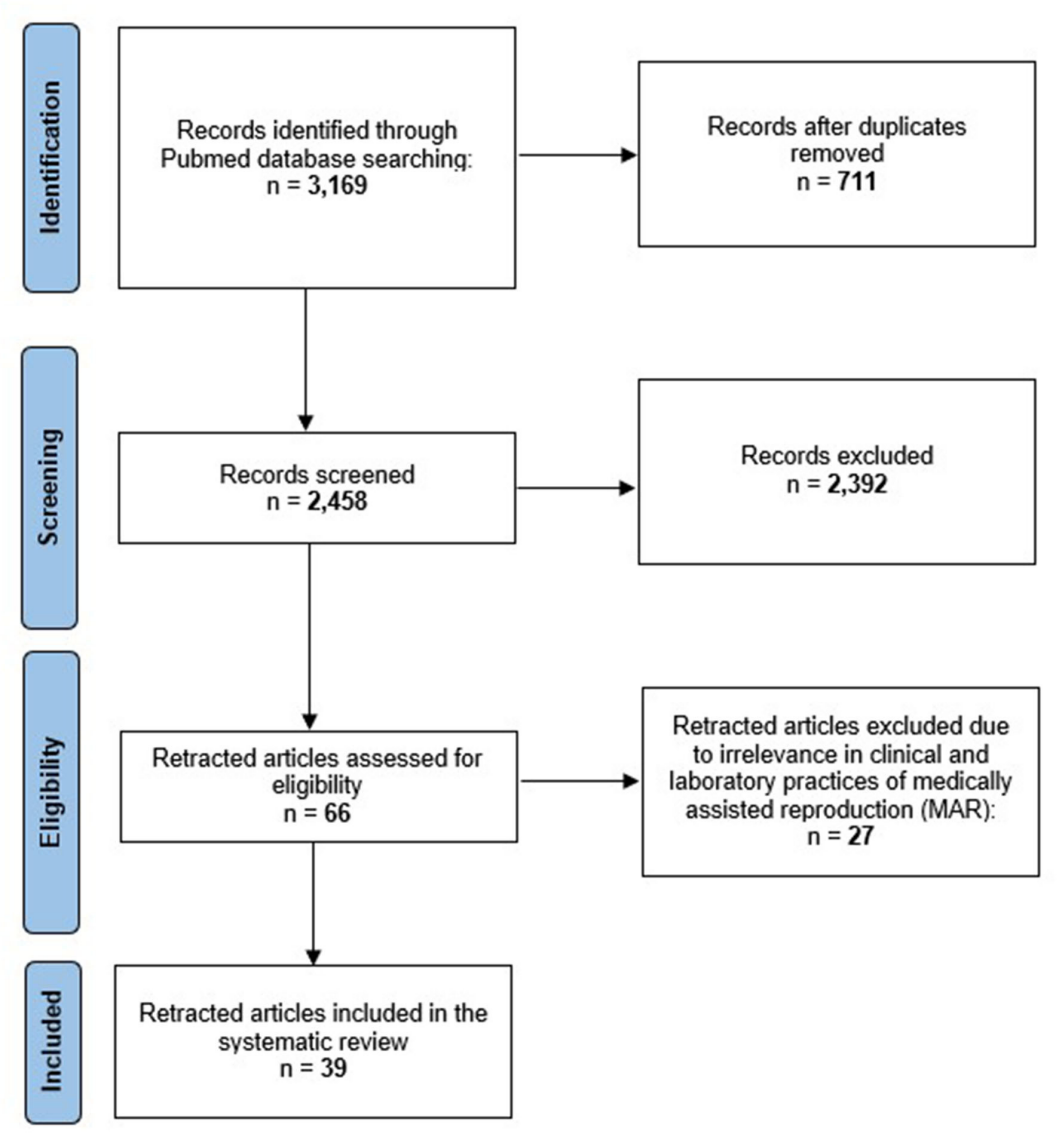
PRISMA flow diagram of our systematic review.

A systematic review was performed in order to identify retracted studies exclusively focused on clinical and laboratory practices related to MAR, and, out of 2,458 items, a total of 2,392 records were excluded after title/abstract screening. Subsequently, 66 manuscripts were identified as potentially eligible. After reading the full texts, *n* = 27 papers were excluded due to irrelevance in clinical and laboratory practices related to MAR, and a total of 39 studies were included in the systematic review (Supplementary Table II). The study flow diagram of the systematic review is represented in Figure 1.

**Figure 2:**
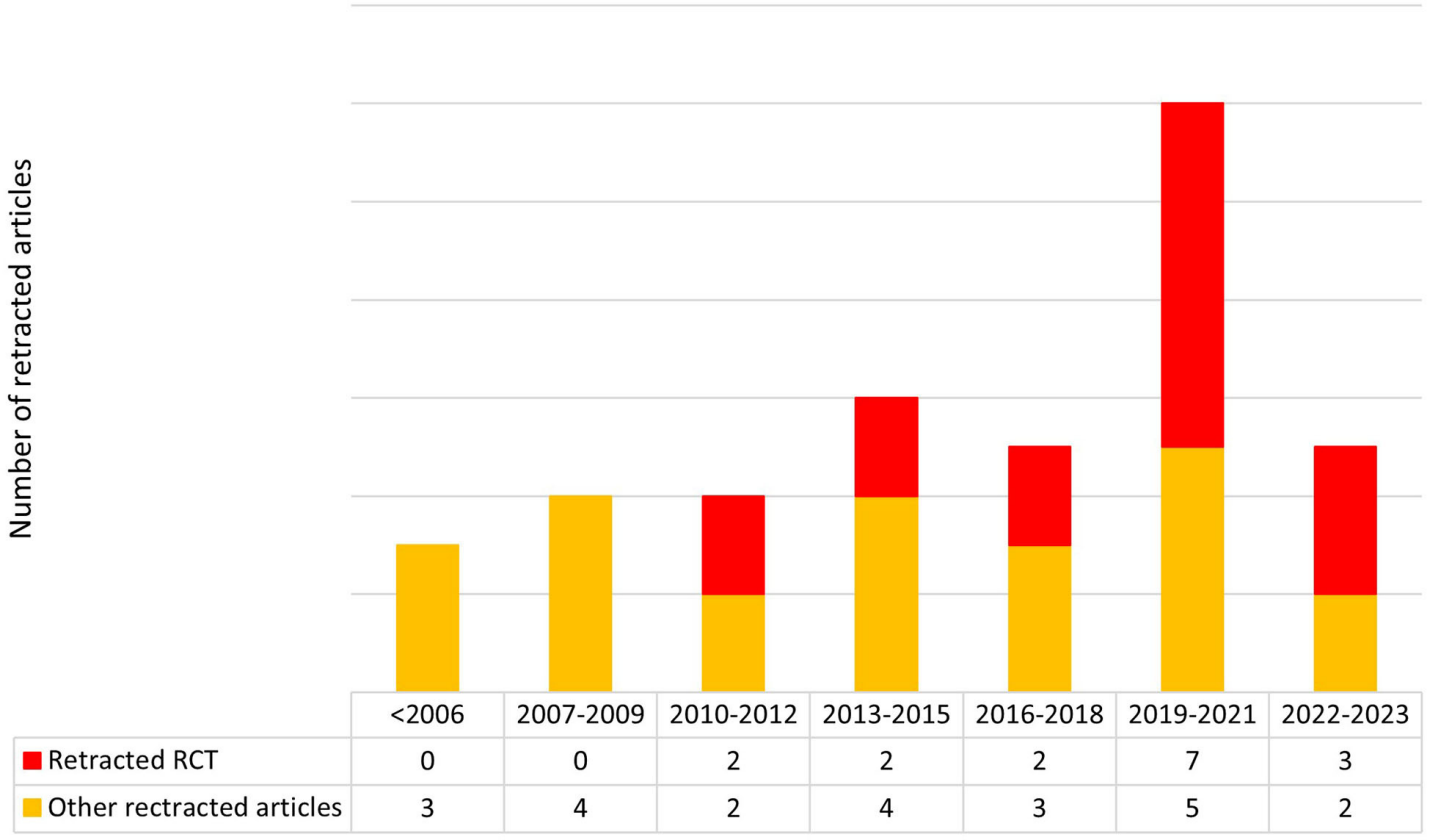
Growth in the number of retracted articles and specifically randomized controlled trials (RCTs) from 1993 to 2023.

Between 1993 and 2023 the number of retracted studies has remained relatively constant, with a noticeable increase observed between 2019 and 2021 (*n* = 12/39; Figure 2).

Among the clinical studies in the field of MAR retracted from the literature, 41.0% were RCTs (*n* = 16), 15.4% were reviews (*n* = 6), 10.3% were retrospective studies (*n* = 4), and prospective studies (*n* = 4), 7.7% were letters (*n* = 3), and case reports (*n* = 3). Two articles were case-control studies while one was a meta-analysis (Table 1).

**Table 1:** Features of medically assisted reproduction retracted articles.

**Study design**	***N*o. of retracted studies**	**%**
Randomized controlled trial	16	41.0
Review	6	15.4
Prospective study	4	10.3
Retrospective study	4	10.3
Letter	3	7.7
Case report	3	7.7
Case control study	2	5.1
Meta-analysis	1	2.6
	39	100.00
	**Median**	**Range**
Impact factor	3.36	0.58–96.22
Time to retraction (years)	2	< 1–12
Article citations	3	0–42

The median IF of journals in which papers were published and retracted was 3.4 (range of 0.58–96.22). The highest and currently available IF of a journal with a retracted study is 96.22 (2021) of the British Medical Journal (BMJ) while the lowest is 0.58 (2021) from Medical Acupuncture. Most of the considered papers were retracted within ~2 years of publication (median, 2; range, < 1–12 years) and the median number of times that a retracted article was cited was 3 (range, 0–42; Table 1). Moreover, the median number of times that a retracted article was cited after their retraction was 2 (range, 0–13). In detail, the 39 MAR retracted papers were found across 23 different journals with their own ethical guidelines.

Egypt recorded the highest number of retractions (*n* = 9), followed by China (*n* = 7), the Republic of Singapore (*n* = 4), Italy (*n* = 3), and Turkey (*n* = 3). For this analysis, we considered only the affiliation of the first author as primary authorship.

Table 2 presents reasons for paper retraction. A total of 35 studies were retracted for a single reason while 4 studies were retracted due to 2 different reasons. The most common reason for MAR paper retraction was plagiarism (13/43; 30.2%), followed by duplicate publication (11/43; 25.6%) and errors in data (9/43; 20.9%). One study was retracted for duplicate publication and error, as well as lack of ethical approval and undeclared conflict of interest, and violation of editorial policy. Finally, in one paper the reason for the retraction was not reported (Table 2).

**Table 2:** Reasons for articles retraction in the field of medically assisted reproduction.

**Reasons for retraction**	**No. of retracted studies**	**%**
Plagiarism	13	30.2
Duplicate publication	11	25.6
Errors in data	9	20.9
Fraud or suspected fraud	4	9.3
Irregular citation pattern	2	4.7
No ethical approval	1	2.3
Undeclared conflict of interest and breach of editorial policy	1	2.3
Compromised peer review process	1	2.3
Not specified	1	2.3

Additionally, examining the retraction notices of all retracted studies revealed that these were mainly issued by the editor and the publisher. Only a single manuscript was not associated with an accessible retraction notice, as shown in Table 3.

**Table 3:** Number of retracted studies listed for those who requested retraction.

**Who retracted?**	**No. of retracted studies**	**%**
Editor	20	38.5
Publisher	13	25.0
Authors	11	21.2
Journal	7	13.5
Not specified	1	1.9

## Discussion

The retraction of a scientific publication is considered a severe penalty for various reasons, including honest mistakes made in good faith, as well as intentional misconduct. Specifically, research misconduct is a rare event due to the low proportion of publications that are retracted. However, many more articles should be retracted, and intentional violation of research integrity principles happens more often than we like to believe (18).

The retraction process is a common procedure that has been documented to occur in various fields, including medical oncology (19), general and plastic surgery (20), dentistry (21), orthopedics radiology (22), and neurosurgery (23), demonstrating that misconduct is observed through all fields of medical research. In obstetrics and gynecology, the number of retracted publications is constantly increasing (10–12).

This is the first study to report an estimate of the citation retraction rate in the field of infertility and fertility care, which was found to be 0.47%. Specifically, within this field, we observed that 0.20% of RCTs published between 1993 and the beginning of 2023 were retracted. Our findings indicate that the proportion of retracted citations is low, but it is important to note that the citation retraction rate reported in this study is an estimate, as it is based on retracted citations among the records identified through the literature search. Due to the impracticality of screening the entire pool of published studies, it may not capture the complete picture of retractions in the field of infertility and fertility care.

MAR represents a crucial aspect encompassing various interventions, procedures, surgeries, and technologies aimed at treating different forms of fertility impairment and infertility. These include ovulation induction, ovarian stimulation, ovulation triggering, all assisted reproductive technology procedures, uterine transplantation, and intrauterine, intracervical, and intravaginal insemination with semen from the husband/partner or donor. The aim of our study was to systematically identify and focus on retracted literature specifically related to MAR, resulting in the inclusion of 39 retracted articles.

The majority of these manuscripts were retracted shortly after their publication and, between 2019 and 2021, the number of retracted papers clearly increased. One plausible explanation for the increase in the number of retractions observed between 2019 and 2021 could be attributed to a greater emphasis on research integrity, publication ethics, and responsible conduct of research that emerged during that period in the field of obstetrics and gynecology, as evidenced by numerous studies (11, 24, 25). However, it should be noted that the number of retractions appears to decrease in 2022–2023, but this could be attributed to the fact that our systematic review only considered retractions until February 2023, resulting in data for only 1 year.

We performed a descriptive analysis to determine the country associated with the most retractions. For this analysis, we considered only the affiliation of the first author as primary authorship is the most noticeable to readers (26). We highlighted Egypt to hold primacy in the number of retractions over other countries. A potential explanation for the higher number of retractions in Egypt could be attributed to the fact that of the nine retracted articles, five were authored by the same person, whose studies raised doubts and prompted further investigation (25).

The main reasons for retraction were plagiarism (appropriation of another's idea/results or copying parts of a previously published article), duplicate publication (published same data more than once), and errors in data (unrealisable or compromised data). Other reasons for retraction included fraud or suspected fraud (data or images manipulation), no ethical approval (articles without ethical approval), undeclared conflict of interest and breach of editorial policy (no conflicts of interest have been declared and the manuscript violated an editorial policy) and compromised peer review process. In some cases, articles were retracted for a combination of different reasons. In line with our findings, Chambers et al. (10) reported plagiarism as the most common reason for retracted publications in reproductive endocrinology and infertility including only seven studies related to this topic in their analysis. It is essential to note that the interpretation of these retraction reasons should be considered within the context of the editorial decisions made. While we cannot provide specific details regarding the impartiality of each editorial decision, we acknowledge the importance of considering the potential impact of editorial biases in the retraction process. Further research and transparency in the editorial decision-making process would be valuable in assessing the unbiased nature of the retractions observed in our study.

RCTs were the most retracted study type. Most of these retractions occurred following the publication of the study by Bordewijk et al. (25) which suggested serious concerns about data integrity in published RCTs of two authors from the same university. Moreover, Li et al. (27) identified at least 20 RCTs in the field of obstetrics and gynecology that were retracted for scientific misconduct. RCTs are considered the capstone of scientific evidence pyramid for the effectiveness and safety of treatments in medicine. RCTs and meta-analyses of RCTs often provide the framework for drafting national and international guidelines while compromised RCTs can lead to the use of unnecessary or even detrimental interventions.

Many concerns arise from the number of citations after retraction occurred. Previous studies found that many retracted articles continue to be cited as if they were still applicable (28) while others observed an immediate effect of retraction on citation rate (29). Our findings demonstrated that MAR articles continue to be cited after retraction, promoting the circulation of erroneous information for prolonged periods. However, it is possible that these studies were cited as an example of problematic and fraudulent research and not to endorse their validity. Further studies are needed in order to examine reasons regarding post-retraction citations.

Retraction notices are signed by authors, journals, editors, and/or publishers. Our study showed that although mainly editors release retraction notices, these are not elaborative and, hence, not insightful.

There are several limitations associated with our study. First of all, we determined the overall citation retraction rate within the field of infertility and fertility care by utilizing the 283 keywords from the Infertility Glossary (15). Due to the substantial denominator of 523,067 articles, we were unable to comprehensively screen all of these studies and consequently calculate the precise retraction rate, specifically for the field of MAR. Furthermore, in our search for retracted articles of MAR, it is important to acknowledge that some articles may have been inadvertently missed during the study search. This could be attributed to the inherent difficulty in understanding whether the topic was inherent to MAR. Additionally, the Retraction Watch website (30) has not been considered due to the high number of keywords used for the study search. Moreover, specific information within the retraction notices as retraction reason and other details often were missing. This may lead to unreliable data. Finally, we acknowledge that the exclusion of non-English articles may introduce a potential bias and limit the generalizability of our findings.

Nevertheless, to the best of our knowledge, this is the first study to explore retractions in MAR field. The inclusion of all keywords from the Infertility Glossary (15) made it possible to identify and include more articles focused on MAR compared to the study performed by Chamber et al. (10). Moreover, registering the protocol of this systematic review allowed reducing research bias, duplication of effort, and resource waste, providing greater transparency.

This systematic review has the definitive aim of drawing attention to research misconduct and raising awareness throughout the MAR scientific community. Kemper et al. (31) highlighted that research in reproductive endocrinology and infertility should be improved due to a high number of studies that are not registered and without accessible protocols. The consequences of disseminating untruthful scientific information and retraction *per se* can be significant. It is the responsibility of the scientific community to ensure proper execution of research that meets specific scientific standards but also to identify misconduct when reviewing the research of peers. Initial screening and subsequent thorough investigation of a manuscript during review is indeed a crucial step before publication and training those researchers who engage in peer review to adhere to instructions and guidelines should be a priority. Likewise, the editorial board of a journal is also significantly responsible for ensuring the absence of plagiarism in all published manuscripts. The advent of online manuscript submission systems has made plagiarism checking less challenging compared to the era of hard copy submissions. In this regard, a reliable web-enabled plagiarism detection tool is a valuable tool for the Editorial Board. Besides authors, editors, and publishers, whose role is to guarantee compliance with COPE guidelines, the institutions affiliated with the research should promote a scientific integrity culture among their researchers by providing training and monitoring of all relevant activities. Considering the collateral negative impact of individual invalid publications on systematic reviews, meta-analyses, guidelines and, eventually, clinical practice, promoting scientific integrity in MAR should be paramount (32).

## Data availability statement

The original contributions presented in the study are included in the article/Supplementary material, further inquiries can be directed to the corresponding author.

## Author contributions

AA conceived the idea for this review. MZ, SMi, and VP performed the literature search. MZ, DM, VP, and SE extrapolated the data. SMi, MZ, SMa, and AA collaborated in writing the first draft of the manuscript. MCo, MCa, and EP contributed to critical revision. All authors critically reviewed the article resulting in a revision of several drafts, read, and approved the final version of the manuscript.

## References

[1] Committee on Publication Ethics (COPE) Guidelines: Retraction guidelines. (2019). Available online at: https://publicationethics.org/sites/default/files/retraction-guidelines-cope.pdf (accessed April 1, 2023).

[2] Nath SB ARTcus SC Druss BG. Retractions in the research literature: misconduct or mistakes? Med J Aust. (2006) 185:152–4. 10.5694/j.1326-5377.2006.tb00504.x 16893357

[3] Steen RG. Misinformation in the medical literature: what role do error and fraud play? J Med Ethics. (2011) 37:498–503. 10.1136/jme.2010.041830 21343631

[4] Wager E Williams P. Why and how do journals retract articles? An analysis of medline retractions 1988-2008. J Med Ethics. (2011) 37:567–570. 10.1136/jme.2010.040964 21486985

[5] Fang FC Steen RG Casadevall A. Misconduct accounts for the majority of retracted scientific publications. Proc Natl Acad Sci U S A. (2012) 109:17028–33. 10.1073/pnas.1212247109 23027971 PMC3479492

[6] Grieneisen ML Zhang M. A comprehensive survey of retracted articles from the scholarly literature. PLoS ONE. (2012) 7:e44118. 10.1371/journal.pone.0044118 23115617 PMC3480361

[7] Moylan EC Kowalczuk MK. Why articles are retracted: a retrospective cross-sectional study of retraction notices at BioMed Central. BMJ Open. (2016) 6:e012047. 10.1136/bmjopen-2016-012047 27881524 PMC5168538

[8] Fang FC Casadevall A. Retracted science and the retraction index. Infect Immun. (2011) 79:3855–9. 10.1128/IAI.05661-11 21825063 PMC3187237

[9] Steen RG Casadevall A Fang FC. Why has the number of scientific retractions increased? PLoS ONE. (2013) 8:e68397. 10.1371/journal.pone.0068397 23861902 PMC3704583

[10] Chambers LM Michener CM Falcone T. Plagiarism and data falsification are the most common reasons for retracted publications in obstetrics and gynaecology. BJOG. (2019) 126:1134–40. 10.1111/1471-0528.15689 30903641

[11] Li W Mol BW. Re: plagiarism and data falsification are the most common reasons for retracted publications in obstetrics and gynaecology. BJOG. (2019) 126:1289. 10.1111/1471-0528.15829 31267647

[12] Bennett C Chambers LM Al-Hafez L Michener CM Falcone T Yao M . Retracted articles in the obstetrics literature: lessons from the past to change the future. Am J Obstet Gynecol MFM. (2020) 2:100201. 10.1016/j.ajogmf.2020.100201 33345918

[13] Page MJ McKenzie JE Bossuyt PM Boutron I Hoffmann TC Mulrow CD . The PRISMA 2020 statement: an updated guideline for reporting systematic reviews. BMJ. (2021) 372:n71. 10.1136/bmj.n71 33782057 PMC8005924

[14] PROSPERO “International Prospective Register of Systematic Review”. Available online at: https://www.crd.york.ac.uk/prospero/ (accessed April 1, 2023).

[15] Zegers-Hochschild F Adamson GD Dyer S Racowsky C de Mouzon J Sokol R . The international glossary on infertility and fertility care, 2017. Fertil Steril. (2017) 108:393–406. 10.1016/j.fertnstert.2017.06.005 28760517

[16] Kovalchik S. RISmed: Download Content from NCBI Databases. R package version 2.3.0 (2021).

[17] R Core Team. R: A Language and Environment for Statistical Computing. Vienna: R Foundation for Statistical Computing (2022).

[18] Li W Gurrin LC Mol BW. Violation of research integrity principles occurs more often than we think. Reprod Biomed Online. (2022) 44:207–9. 10.1016/j.rbmo.2021.11.022 34974962

[19] Pantziarka P Meheus L. Journal retractions in oncology: a bibliometric study. Future Oncol. (2019) 15:3597–608. 10.2217/fon-2019-0233 31659916

[20] Gaudino M Robinson NB Audisio K Rahouma M Benedetto U Kurlansky P . Trends and characteristics of retracted articles in the biomedical literature, 1971 to 2020. JAMA Intern Med. (2021) 181:1118–21. 10.1001/jamainternmed.2021.1807 33970185 PMC8111562

[21] Samuel S Cherian JM Thomas AM. Comprehensive analysis of retracted publications in dentistry: a 23-year review. Int J Dent. (2020) 2020:8881352. 10.1155/2020/8881352 33424973 PMC7781686

[22] Bolboacă SD Buhai DV Aluaṣ M Bulboacă AE. Post retraction citations among manuscripts reporting a radiology-imaging diagnostic method. PLoS ONE. (2019) 14:e0217918. 10.1371/journal.pone.0217918 31194762 PMC6563977

[23] Wang J Ku JC Alotaibi NM Rutka JT. Retraction of neurosurgical publications: a systematic review. World Neurosurg. (2017) 103:809–14. 10.1016/j.wneu.2017.04.014 28412480

[24] Liu Y Thornton JG Li W van Wely M Mol BW. Concerns about data integrity of 22 randomized controlled trials in women's health. Am J Perinatol. (2023) 40:279–89. 10.1055/s-0041-1727280 34005825

[25] Bordewijk EM Wang R Askie LM Gurrin LC Thornton JG van Wely M . Data integrity of 35 randomised controlled trials in women' health. Eur J Obstet Gynecol Reprod Biol. (2020) 249:72–83. 10.1016/j.ejogrb.2020.04.016 32381348

[26] Venkatraman V. Conventions of Scientific Authorship. (2010). Available online at: https://wwwscienceorg/content/article/conventions-scientific-authorship-0 (accessed April 1. 2023).

[27] Li W Bordewijk EM Mol BW. Assessing research misconduct in randomized controlled trials. Obstet Gynecol. (2021) 138:338–47. 10.1097/AOG.0000000000004513 34352811

[28] Pfeifer MP Snodgrass GL. The continued use of retracted, invalid scientific literature. JAMA. (1990) 263:1420–3. 10.1001/jama.263.10.1420 2406475

[29] Furman JL Jensen K Murray F. Governing knowledge in the scientific community: exploring the role of retractions in biomedicine. Res Policy. (2012) 41:276–90. 10.1016/j.respol.2011.11.001

[30] Retraction Watch Database. Available online at: https://retractionwatch.com/ (accessed April 1, 2023).

[31] Kemper JM Rolnik DL Mol BWJ Ioannidis JPA. Reproducible research practices and transparency in reproductive endocrinology and infertility articles. Fertil Steril. (2020) 114:1322–9. 10.1016/j.fertnstert.2020.05.020 32771255

[32] Fauser BC. Protecting data integrity in reproductive medicine. Reprod Biomed Online. (2022) 44:205–6. 10.1016/j.rbmo.2021.12.005 35065911

